# Analysis of Productivity Improvement Act for Clinical Staff Working in the Health System: A Qualitative Study

**DOI:** 10.5539/gjhs.v8n2p106

**Published:** 2015-06-11

**Authors:** Leila Vali, Seyed Saeed Tabatabaee, Rohollah Kalhor, Saeed Amini, Mohammad Zakaria Kiaei

**Affiliations:** 1 Environmental Health Engineering Research Center, Kerman University of Medical Sciences, Kerman, Iran; 2 Health Information Management Research Center, Hormozgan University of Medical Sciences, Bandar Abbas, Iran; 3 Social Determinants of Health Research Center, Qazvin University of medical sciences, Qazvin, Iran; 4 Health Services Management Department, School of Public Health, Qazvin University of Medical Sciences, Qazvin, Iran; 5 Research Center for Health Services Management, Institute for Futures Studies in Health, Kerman University of Medical Sciences, Kerman, Iran

**Keywords:** productivity improvement act, health care system, clinical staff, Iran

## Abstract

**Introduction:**

The productivity of healthcare staff is one of the main issues for health managers. This study explores the concept of executive regulation of Productivity Improvement Act of clinical staff in health.

**Methods:**

In this study phenomenological methodology has been employed. The data were collected through semi-structured interviews and focus group composed of 10 hospital experts and experts in human resources department working in headquarter of Mashhad University of Medical Sciences and 16 nursing managers working in public and private hospitals of Mashhad using purposive sampling. Findings were analyzed using Colaizzi’s seven step method.

**Results:**

The strengths of this Act included increasing spirit of hope in nurses, paying attention to quality of nursing care and decreasing problems related to the work plan development. Some of the weaknesses of Productivity Improvement Act included lack of required executive mechanisms, lack of considering nursing productivity indicator, increasing non-public hospitals problems, discrimination between employees, and removal of resting on night shifts. Suggestions were introduced to strengthen the Act such as increased organizational posts, use of a coefficient for wage in unusual work shifts and consideration of a performance indicator.

**Conclusion:**

The results may be used as a proper tool for long term management planning at organization level. Finally, if high quality care by health system staff is expected, in the first step, we should take care of them through proper policy making and focusing on occupational characteristics of the target group so that it does not result in discrimination among the staff.

## 1. Introduction

The main basis of each organization is its human resource. The role of human resource is not only in the organization level, but also its role has expanded from a small economical unit to the national level (Pohjola, 2010). The relationship between human resources and performance has been widely researched over the last twenty years, especially in the USA (Arthur, 1994; Osterman, 1994; Huselid, 1995; MacDuffie, 1995; Koch & Mcgrath, 1996) and in the Great Britain (Guest & Hoque, 1994; Mcnabb & Whitfield, 1997; Guest, 1999), where a number of research studies lead us to understand this relationship. The main outcome of these studies has been the recognition that human resource management has considerable impact on organization’s competitive capacity (Delery & Doty, 1996; Golinelli et al., 2005).

The low individual productivity in the governmental agencies has caused to pay attention to the human resource improvement. One of the important ways for human resource improvement is their assessment. Organization management through employees performance assessment decide about delegating tasks and responsibilities, promotion, mobility and transferring, and training of staffs. Pay attention to the human resource is one of the key strategies to increase organization efficiency and productivity (Bertschek et al., 2009).

Among the organizations, management and performance improvement in hospitals has a special place, because hospital is the most important center for providing diagnosis, treatment and rehabilitation services. Hospitals also have more varied and higher employees organizational positions than other organization (Ashby et al., 2005). The productivity of healthcare staffs is one of the main current issues and is one of the most significant challenges that health sector managers face it (Mardani et al., 1999). Productivity of this group is one of the biggest concerns for health care organizations (Mc-Neese-Smeet, 2001). Despite the key importance of productivity, this phenomenon has rarely been studied in nursing. Similarly, if productivity has been considered, it has been in terms of the materials from industry and economic theories but outcomes of nursing practice have not been considered (Eastaugh, 2007; Jordan, 1994; Vincent & Ravinder, 1989).

In order to increase efficiency and effectiveness of clinical staffs in healthcare system in both public and private sectors, Productivity Improvement Act (PIA) was passed by the parliament in 2009 and its executive regulation passed by cabinet in 9 articles and 2 notes in the same year. PIA is the first legal document regarding to the issue of productivity for clinical staffs in health care system. Clinical staffs that include nurses, healthcare workers, helping healthcare workers, midwives, technicians and experts in operating room, anesthesia and medical emergency providing services directly to hospitalized patients, benefit from PIA. By implementing this plan, work hour of target group will be decreased from 44 hours per week to 36 hours based on three factors of experience, hard work and shift work. Work night shifts and weekend work are also calculated based on work coefficient of 1.5. Employees subject to the Act are not allowed to work more than twelve consecutive hours and there is one hour for shifting work at the end of the shift. Target group are not allowed to rest and sleep during night shifts and holidays. In psychiatric and burn departments, other than the target group, the mental experts, social workers and occupational therapists also benefit from one extra month paid leave in addition to primary one month paid leave; this leave cannot be stored and must be used during the year (Ministry of Health and Medical Education, 2009). There are approximately 940 hospitals in Iran where the largest number of hospitals affiliated to the Ministry of Health and Medical Education (MOHME). The number of hospital beds is approximately 116,689. (Ministry of Health and Medical Education, 2014) Distribution of hospital beds is shown in Table 1. This study explores the concept of executive regulation of Improvement Productivity Act of clinical staffs in health system. Description of the regulations of this Act can help policy makers to identify its strengths and weaknesses.

**Table 1:** Frequency of hospitals and beds in Iran-2014

Hospital’s Affiliation	Hospital NO	Bed NO
Ministry of Health and Medical Education	584	79291
Private Sector	151	14187
Social Security Organization	74	10376
Military Hospitals	53	4965
Charity Hospitals	30	3274
Others	48	4596
Total	940	116689

## 2. Method

In this qualitative study, the phenomenological methodology has been employed. Focus group discussion has been used for data collection. Two separate groups of experts were used to gain insights. One group composed of 10 hospital experts and experts in human resource departments working in headquarter of Mashhad University of Medical Sciences and other group included 16 nursing managers working in public and private hospitals of Mashhad which were selected using purposive sampling. The discussion group questions asked by the facilitator were focused on three areas: advantages, disadvantages and suggestions of clinical staffs working in Iran health system about the PIA. Each focus group discussion took around 1.5- 2 hours. At the opening of the session the facilitator explained about the purpose of the study, voluntary participation and the confidentiality of the contents expressed by the participants. Consent was obtained from all participants to record their voice. Then each participant explained his own viewpoints. After the session the audio file was heard and content analysis was used to derive the results.

Based on Guba and Lincoln, reliability and validity are based on the four main criteria (truth value, applicability, consistency and neutrality) (Polit, 1993). In this study, these criteria have been met through confirming the statements by participants (actual value), selecting various participants (applicability), the similar response by the participants to the same questions in different formats (consistency) and avoiding any prejudice and bias during the interview (neutrality). The data were analyzed using seven-step Colaizzi’s method (Saneie & Nikbakht, 2004). After conducting interviews and writing the recorded information on paper, all the items were read carefully and most important terms (including rich concepts regarding PIA) excluded to understand the participants` ideas and obtain deeper understanding of the concepts. In the next step, the meaning of each phrase was explained and the meanings were written down in terms of codes. Then, the codes were organized in categories and these categories were compared with the participants` main descriptions to authenticate them. Next, the results were combined as complete description of the experience of PIA enforcement by the participants and then were revised in order to obtain clear and unambiguous concepts. Finally, to determine validity, the results referred to the participants and they confirmed reliability of the results in the final interview.

## 3. Results

In total, 16 nursing managers and 10 hospital experts and experts in human resources department were interviewed. Participants included 18 women (69%) and 8 men (31%) with a mean age of 41/2 ± 6/4 years and average years of experience 19 ± 7.6 years. The findings were divided into three major themes and 16 subthemes which are provided in Table 2.

**Table 2:** Themes and subthemes derived from focus group discussions

Themes	Subthemes
Strengths of the productivity improvement Act	-Increasing spirit of hope in nurses -Paying attention to quality of nursing care -Decreasing problems related to the work plan development
Weaknesses of the productivity improvement Act	-Lack of required executive mechanisms for human resources recruitment -Lack of considering nursing productivity measurement indicator -Lack of considering the livelihood condition in the Act target group - Increasing non-public hospitals problems - Discrimination between employees -Lack of considering the past Acts -The removal of resting on the night shift
Suggestions for strengthening the productivity improvement Act	-Increasing organizational posts - Using coefficient for wage in unusual work shifts -Generalizing floating coefficient to all personnel - Considering the performance indicator -Enforcing the tariff setting Act for nursing services

A) Strengths of the productivity improvement Act

1) Increasing spirit of hope in nurses

Many of interviewees stated the understanding that their performance is considered by officials and legislators has a positive impact on staffs morale and passing such Acts encourages providing effective services for patients. In this regard, one of the nursing managers expressed that “paying attention to nurses’ productivity within the Act is an initiative that has positive impact on the morale of staffs and increase their hope to work more efficiently”.

2) Paying attention to quality of nursing care

Some respondents stated that the above mentioned Act will increase the quality of nursing cares. In this regard, one nursing manager expressed that “… the main purpose of this Act is paying attention to the quality of nursing care so that the probability of nursing errors are reduced through reduction of nurses work time, reduction of mandatory overtime and working hours no more than 12 hours a day…”. Quality of nursing care is defined as the nursing response to the spiritual, social, emotional and psychosocial needs of patients in order to maintain and improve patient’s health (Tafreshi, 2007). Similarly, a number of interviewees stated that devoting time to change current affairs in the department based on the Act causes to improve the quality of nursing care. In this regard, one nursing manager expressed that before the Act, delivery and change of affairs in the department were not being done properly, but now nurses do it with greater precision and patience, because one hour has been devoted to do so.

3) Decreasing problems related to the work plan development

Development of nursing work plan in order to meet nurses, patients and hospital needs always has been a challenging subject for nursing hospital managers. Problems such as shortage of manpower and lack of willingness to participate in unconventional shift and lack of presence in intensive departments are some of the main problems related to the work plan development (Huber, 2000). In this regard one of the nursing managers stated that “…previously, night shifts and holidays were only for new staffs but through implementing this Act and calculating 1.5 times more paid for each night and holiday shifts, the experienced staff are now more willing to work in night shifts and holidays…”. In this regard one of the interviewees pointed to other various points such as motivation of nurses to work in certain sectors (such as burns or psychic) or incentives to not apply for early retirement. One respondent, for example, stated that “…few people are willing to work in burn and psychic departments, but fortunately we hope that the problems in these departments be decreased through applying one month extra and mandatory leave…” (One of the nursing managers).

B) Weaknesses of the productivity improvement Act

1) Lack of required executive mechanisms for human resources recruitment

Given the calculation of 1.5 times for unconventional work shifts and lack of permission to work for more than 80 hours per month and also considering one month mandatory leave in addition to one month paid leave for staff working in burn and psychic departments, the need for human resources to cover work shifts will consequently increase. In this regard, some respondents considered the lack of required executive mechanisms for human resources required for this Act as a weakness. One expert in human resources planning, for instance, stated that “… an estimate of the increase in staffing was needed before implementing the Act in order to add hospitals` job positions, but that did not happen in practice…”

2) This Act was only in the benefit of development of hospitals departments

Concurrent with the implementation of the Act, many hospitals were developing new or current departments based on the rationing services plan; this issue increased the problems related to implementing of PIA, because most of the devoted employees to implement this Act were recruited in the new and developed departments. In this regard, one respondent stated that”…recovery of the inpatient departments was expected through these numbers of nursing staffs entered into the hospitals, but unfortunately because of not increasing the hospitals` job positions for the new and developed departments, most of these staffs were recruited in the new and developed departments…” (one of the nursing managers).

3) Lack of considering nursing productivity measurement indicator

A number of respondents pointed out that merely developing and implementing the Act cannot ensure the clinical staff productivity improvement. In this regard, an expert in hospital affairs stated that “… when we are not able to measure the quality of a service, with no policy and strategy we cannot claim that we could improve it. When there is no indicator to measure the quality of nursing care, how this Act can claim that the productivity has been improved, just the reduction of work time is not a scale”. Another hospital affairs expert also expressed that “when our hospital infection rate is reported less than the world level, when pharmaceuticals errors are not reported, when the patient`s partner has to be silent and does not complaint the inappropriate treatment by the nurse due to fear of lack of care, how we want to improve the quality of care through this Act”.

4) Lack of considering the livelihood condition in the act target group

Salary and wage gap in the health system is very high compared to other sectors. Although the nursing profession has been reported as one of the toughest jobs, they receive one of the lowest salaries and wages among the employees. So, some of them inevitably have to work long shifts in hospitals. Undoubtedly, this issue plays an important role in decreasing the nurses` ability to provide high quality care (Lu, 2005). One of the nursing managers expressed that “…given that currently 30% of employees are paid for 175 overtime hours and other 70% are paid for almost 100 overtime hours, considering 80 overtime hours will definitely put the employees` livelihood condition in trouble…”. Some interviewees believed that in order to paying attention to nursing staff livelihood condition, the act of setting tariff for nursing services should be passed before implementing the Act so that one interviewee stated that “…Tariff Act is an incentive for improving the nurses` livelihood conditions and it would be better Tariff Act be passed before the productivity improvement Act. Passage Tariff Act facilitates the productivity Act implementation …” (one of the nursing managers).

5) Increasing non-public hospitals problems

Some interviewees, especially those working in private hospitals emphasized on the problems of hospitals because of implementing this Act. One of the nursing managers in private sector, for example, expressed that “…the employees` salary in the public sector is financed from general government budget, but the increase of human resources exacerbates the condition of private and charity hospitals which are in break-even point and the government does not help them…”.Through implementing this Act, many of Act target group employees in private and charity hospitals were absorbed by the public hospitals and this issue put the non-public hospitals in trouble so that one participant stated that “…most experienced and trained nurses in the private sector have been absorbed by the public sector in a short period time so that some private hospitals` departments have been shut down due to human resources shortage …” (one of the nursing managers- the private sector).

6) Discrimination between employees

It can be inferred from the interviews concepts that this Act has caused discrimination between hospital staffs who directly deals with patient since within this Act clinical staffs in health system is limited to staff of nursing, midwifery operating room, anesthesia and medical emergencies and other clinical staff such as staffs in diagnosis and rehabilitation departments have been ignored. They also pointed to the effects of this discrimination which is decrease of teamwork. In this regard, one of the experts in hospital affairs expressed that “…the treatment process often begins in diagnostic departments such as laboratory, radiology and so on and the employees working in these departments directly and/or indirectly provide services to patients. Risks and hardness of the work of this group of staffs, if not more, certainly not less than the target group but their names have not been mentioned in the Act…”. The interviewees also believed that lack of considering the teamwork by the Act has had negative effect on other hospitals plans such as clinical governance so that another expert in hospital affairs stated that “…low motivation and lack of spirit of teamwork among the staffs is one of the challenges of implementing clinical governance system and current problems in the organizational culture in hospitals that vary depending on hospital, and the productivity Act strongly strengthens this culture due to lack of paying attention to teamwork…”.

7) Lack of considering the past Acts

In this regard, some interviewees emphasized on scoring according to job characteristics and believed that one job characteristic should be considered once for one score and the scores considered previously for that characteristic should not be ignored. In this regard, one expert in hospital affairs stated that “…once it is said that nurses should be paid extra for unconventional shift work, next year it is said that because nurses have unconventional shift work, they should take time off work for evening and night shifts, then it is said that because nurses have unconventional shift work, they should be paid using 1.5 coefficient. In sum, I promise in the next years the nursing system organization will get more advantages from the Ministry of health. There is the same story in regard with hard work…”

8) The removal of resting on the night shift

Some participants stated that by removing rest time on night shift, in practice this Act has provided possibility to increase errors and decrease the quality of nursing care. In this regard, one interviewee expressed that “…a car on the road cannot move continuously for 12 hours without rest. How can we expect nurses to work 12 hours without a moment of rest…” (one of the nursing managers). Similarly, some other respondents pointed to other various issues, one of them noted that “…it is said that in a certain city the rest rooms in inpatients wards have been removed, so the nurses who previously used to rest there, now sleep on the desks or in patients` room …” (one of the nursing managers).

C) Suggestions for strengthening the PIA

According to the discussed positive and negative effects of PIA, the interviewees were asked to provide suggestions in order to facilitate the implementing of the Act. The interviewees` suggestions have been outlined as below:

1) Increasing organizational posts

The respondents considered increasing organizational posts and issuing work permit as the main and most significant factors to more efficient implementation of the Act. One of the interviewees, for example, stated that “… adequate human resource ensures PIA implementation…” (one of the nursing managers). Another interviewee also expressed that “this Act is applicable only through attendance of fixed and permanent forces, the temporary forces, which are today but not tomorrow, just have a palliative role (one of the nursing managers).

2) Using coefficient for wage in unusual work shifts

According to the livelihood condition of staff and also low capacity of human resource absorption by the university due to lack of adequate organizational posts in hospitals formation and given that financial payments are one of the most significant and efficient tools for motivating the human resources, many interviewees believed that financial incentives may facilitate the implementation of this Act. In this regard, one participant stated that “…It was better to apply coefficient to wage for unconventional work shifts instead of for days-off and night work shifts because by doing so, the livelihood condition of the target group would be considered and also no more human resources would be added to the system for this issue…” (one of the experts in human resource planning).

3) Generalizing floating coefficient to all personnel

Many respondents suggested fair treatment and removing discrimination between all clinical staff in healthcare system and believed that benefits related to the part of the Act regarding job characteristics (for example, working in unconventional shift) which are devoted to the target group, should be devoted to other staffs having that job characteristic. In this regard, one interviewee expressed that “… it seems better to generalize floating coefficient to all shift-work personnel in order to remove the sense of discrimination among hospitals` employees…” (one of the experts in human resource planning).

4) Considering the performance indicator

The interviewees believed that using performance indicators before and after the Act may help to address the effectiveness of the Act, so they suggested that it would be better to measure performance indicators precisely before complete implementation of the Act, so that the weaknesses and strengths of the Act become obvious, and more efficient effort can be made to eliminate the defects and strengthen the Act. One of the nursing managers, for example, stated that “…defining indicator to measure the performance is very effective in order to identify weaknesses and strengths of this Act provisions. …”.

5) Enforcing the tariff setting Act for nursing services

The interviewees considered the passage of the Act of tariff setting as a prerequisite and complementary for the PIA and believed that this Act, more than other Acts, is needed by nursing personnel. One of the participants, for example, stated that “…in addition to help us to get our rights, the setting tariff Act may very effective in implementing the productivity improvement Act.…”. (one of the nursing managers). In this regard, another respondent expressed that “…if setting tariff Act was passed before this Act, maybe there was no need to this Act …”

## 4. Discussion

The findings of the study show the viewpoints, suggestions and existing experiences of the nursing managers and the experts in human resources field and hospitals affairs regarding positive and negative aspects of “Productivity Improvement Act for clinical staff working in the health system” according to the country healthcare system characteristics.

The participants pointed to the increasing of self-confidence and work morale among the nurses. They believed this Act signifies the officials` attention to the nursing profession and also attention to the quality of nursing care. High morale, is a sign of an appropriate organizing and management, and is considered as a measure of organization progress due to the proper management (Jashnizadeh, 2006).

The participants of the study considered this Act in line with the main mission of the health system to improve the quality of care and emphasized that reducing work time, considering a specified ceiling on overtime work and certain time for switching shifts, all signify the officials` attention to the consequence of nursing care for patients. Williams in his study explored the nurses` viewpoints on the quality of nursing care and concluded that the human resource shortage, increase of consecutive shifts and working mandatory overtime have resulted in increasing nurses` stress and decreasing the quality of nursing care and productivity (Williams, 1998).

Currently, in Iran, work shifts delivery is reported orally and using kardex and without following a single directive and most nurses relying on the record and kardex, flinch to deliver shift work (Rytterstrom, 2009). Besides, lack of correctly identifying patients at medical centers leads to wrong health care and actions such as in process of medications, surgery, blood transfusion, laboratory practices and delivering a baby to the mother and family. Given the harm and damage resulting from this issue, it is essential that reduction and, if possible, elimination of errors due to lack of correctly identification of patients at the time of delivering treatment, diagnostic and care services, be a central point in improvement of patient safety programs in hospitals (Ministry of Health and Medical Education, 2009).

Work shift is the main condition in the field of health services delivery and related problems such as unwillingness of experienced staff to work in night shifts and holidays and recruiting less experienced nurses and novices, lead to the reduction of the quality of care and the increase of patients` dissatisfaction (Sean, 2002). Applying coefficient for night and holidays shifts has been considered as one of the strengths of the Act from many participants` point of view.

The interviewees considered lack of estimation and allocation of required human resources as the most significant elements in regard with lack of required mechanism for implementing the Act. Smeet study showed that human resources productivity is changed as a result of the pattern and organization of human resources within hospitals (Mc-Neese-Smeet, 2001). Similarly, in another study, 37% of the participants considered over workload and inappropriate organization of human resources at the time of shortage as obstacles to productivity (Eastaugh, 2002).

Lack of paying attention to quantitative measurement and nursing care performance indicators as care consequences in order to compare the status before and after the Act implementation has been considered by many respondents as one of the Act weaknesses. If we cannot measure the quality and performance, we never can do something in regard with whether the quality and performance are satisfying or should be improved (Cromwell & Pope, 1989).

While many participants considered putting a ceiling for overtime as a strength point, some other pointed to livelihood problems for the staff resulting from this issue. Nasiripour in his study concluded that our employees do still deal with their first needs i.e. physiological needs and 70.8% of the studied employees considered salary and wage and job security as two most effective deterrents to improve their performance (Nasiripour et al., 2013). One of the characteristic for a proper system of salary and wage is to be reasonable and fair according to the employees` viewpoints and their economic situation (Saadat, 2008).

Almost all of the participants working in the private sector expressed that increasing the nursing human resources for implementing Productivity Improvement Act, has increased the problems of private hospitals and unfortunately no plan has been developed and implemented for compensating the overhead costs resulted from this program in the private health sector. Similarly, financial burden has been imposed to the private hospitals due to the Act implementation and increase of human resources number (Gavgani, 2011).

Another point noted by the interviewees was that the Act is based on guild interests which results in discrimination between employees within a system. Toll believes that fair treatment with employees is a significant factor causing their commitment to organization. Employees who perceived have been treated fairly and free from any discrimination are more likely to contribute to social behaviors which benefit their organization (Toll, 2006).

Lack of paying attention to teamwork resulted from discrimination between the employees was another weakness point emphasized by the participants. Effective communication and teamwork are essential in order to provide high quality care. Lack of paying attention to effective communication and teamwork in hospitals is the main reason for unintentionally harming patients (Leonard, 2004). Williams (1998) in his study emphasized on this issue and expressed that concentrating on quality is not provision of high quality nursing care by an individual, but by a team which has been devoted to provide care to patient from admission to discharge (Williams, 1998).

Lack of considering the past acts were another weakness point expressed by some participants. They believed that it is necessary that in proposing and passing Acts, the officials pay attention to the issue that scores for job characteristics are not considered periodically. According to the clauses 3 and 8 of Article 54 in administrative and employment regulation for non-academic staff working in the Universities of Medical Sciences and Health Services throughout the country, extra payments have been considered for hard work conditions and shift work (Mobaraki, 2012).

In many participants` viewpoints, elimination of the rest time in night shift has caused reduction of nurses` concentration and this issue will affect both nurses` health in general, patient safety and quality of nursing care, in particular. Hojati in his study concluded that there was a statistically significant relationship between sleeplessness and nurses` general health, and general health decreases along with increasing the effects of sleeplessness (Hojati, 2009). Definitely, nurses who do not enjoy good health are not able to provide proper care, such as physical and psychological support to patients. This raises the risk of medication errors and occupational hazards which eventually its consequences involve patients and nurses (Soleimany, 2007).

Attempt for increasing organizational posts and issuing work permit along with estimating required human resources and increasing the number of nursing cadre were some of suggestions proposed by the majority of the interviewees in order to implement the Act efficiently. Dehghani-Nayeri emphasized on this issue and expressed that factors such as workload imbalance and lack of considering nurse patient ratio have a negative impact on productivity (Dehghan, 2005).

Similarly, given that financial motivation is still considered as one of the most important factors in motivating the employees (Herzberg), applying coefficient to wage rates for night work and holiday shifts was another suggestion proposed by some participants, since this issue increase the employees motivation and improve their livelihood condition. Use of nursing performance evaluation indicators was one of the suggestions proposed by a number of participants; they believed that this issue may be effective in identifying the Act strengths and weaknesses. Indicators are scales which express qualitative characteristics in terms of quantities and make them measurable and evaluable (Pazargari, 2003). The main aim of identifying indicators (in any type of identification and classification) is to obtain an evaluation system that is able to measure the quality of nursing care (Brooten, 2004). Although many attempts have been made to define the performance indicators to ensure the quality of nursing care, there are still many challenges regarding this issue. Differences in definitions and methods of identification and classification of the indicators have caused no general agreement and consensus in determining nursing care indicators (ZagheriTafreshi, 2007). The enforcement of the nursing care tariff Act passed in July 2007, has come to naught and the findings of the study strongly suggest that the implementation of this Act may make the nurses` payment reasonable and performance-based and this in turn can increase the motivation and also resolve concern associated with nursing community economic condition.

## 5. Conclusion

Although, almost all studies have considered the productivity equal to efficiency which is quantitative indicator for productivity, the Act trustees and proposers of the Act aim to consider qualitative indicators (effectiveness) since many of their advantages not only benefit patients and improve the quality of care but also maintain and improve the quality of the target group employees life.

Before any move to increase productivity, it should be emphasized on an adequate identification of the current status and appropriate prioritization of actions to prevent any discrimination between different employees of a system who serve within a team. To be aware of the increase of productivity, it should be measured within the various indicators in the given periods of time. To determine the effectiveness of the efforts for improving the productivity, the productivity indicators should be measured based on a reasonable and systematic system. The results of productivity indicators measurement can be used as a proper tool for planning long-term management at organization level. Finally, if we expect the employees working in health care system provide high quality care

to patients the attitude must be created in them that policy makers and decision makers also take care of them in the best way.
